# Sensor-Based Cyber Risk Management in Railway Infrastructure Under the NIS2 Directive

**DOI:** 10.3390/s25237384

**Published:** 2025-12-04

**Authors:** Rafał Wachnik, Katarzyna Chruzik, Bolesław Pochopień

**Affiliations:** Department of Transport and Computer Science, WSB University, 41-300 Dąbrowa Górnicza, Poland; kchruzik@wsb.edu.pl (K.C.); bpochopien@wsb.edu.pl (B.P.)

**Keywords:** railway cybersecurity, NIS2 Directive, sensor networks, IoT, FMEA, anomaly detection, machine learning, risk management, critical infrastructure

## Abstract

This study introduces a sensor-centric cybersecurity framework for railway infrastructure that extends Failure Mode and Effects Analysis (FMEA) from traditional reliability evaluation into the domain of cyber-induced failures affecting data integrity, availability and authenticity. The contribution lies in bridging regulatory obligations of the NIS2 Directive with field-layer monitoring by enabling risk indicators to evolve dynamically rather than remain static documentation artefacts. The approach is demonstrated using a scenario-based dataset collected from approximately 250 trackside, rolling-stock, environmental and power-monitoring sensors deployed over a 25 km operational segment, with representative anomalies generated through controlled spoofing, replay and injection conditions. Risk was evaluated using RPN scores derived from Severity–Occurrence–Detectability scales, while anomaly-detection performance was observed through detection-latency variation, changes in RPN distribution, and qualitative responsiveness of timestamp-based alerts. Instead of presenting a fixed benchmark, the results show how evidence from real sensor streams can recalibrate O and D factors in near-real-time and reduce undetected exposure windows, enabling measurable compliance documentation aligned with NIS2 Article 21. The findings confirm that coupling FMEA with streaming telemetry creates a verifiable risk-evaluation loop and supports a transition toward continuous, evidence-driven cybersecurity governance in railway systems.

## 1. Introduction

The ongoing digitalization of railway transport has resulted in a rapid expansion of sensor-based systems, which continuously monitor infrastructure condition, train movement, energy consumption, and environmental parameters. These sensors, interconnected through Internet of Things (IoT) networks and supervisory control systems (SCADA), form the foundation of modern railway operations. They enable predictive maintenance, traffic optimization, and real-time safety monitoring. However, the same interconnectedness also introduces new vulnerabilities. A compromised sensor node, falsified measurement, or disrupted data stream can propagate through operational systems, affecting traffic control, rolling stock safety, and signaling integrity [1,2,3,4].

In this context, cybersecurity and data integrity have become essential prerequisites for safe and resilient railway operation. The growing reliance on distributed sensor networks, edge devices, and cloud-based analytics transforms traditional railway infrastructure into a cyber–physical system, where the distinction between digital and physical safety is blurred. Consequently, ensuring cybersecurity at the sensor and network level is no longer a purely technical matter but a critical requirement for transport continuity and public safety [5,6,7]. 

Within the European Union, the NIS2 Directive (Directive (EU) 2022/2555) defines new obligations for operators of essential services, including railway transport [5,6,7,8,9,10]. The Directive mandates a high common level of cybersecurity by emphasizing risk management, monitoring, and the continuous evaluation of control effectiveness. For the railway sector, these obligations apply not only to corporate IT systems but also to operational technology (OT) and embedded sensor networks responsible for monitoring tracks, vehicles, and signaling equipment.

While the NIS2 Directive outlines ten categories of cybersecurity measures, two are particularly relevant for sensor-based railway systems:

- Criterion 1: Risk analysis and information system security policy, and
- Criterion 6: Policies and procedures for assessing the effectiveness of risk management measures.

These two provisions jointly form a continuous improvement loop. Criterion 1 defines how risks, including those originating from sensor networks, are identified, prioritized, and mitigated. Criterion 6 ensures that these risk controls are periodically evaluated and adjusted to address evolving cyber threats. In practice, fulfilling these obligations requires methods capable of linking technical vulnerabilities of sensors and IoT systems with organizational risk governance and compliance monitoring.

However, most existing research and industrial practice in railway cybersecurity focus on incident response, supply chain security, or high-level governance models, with less attention given to the sensor-level risk mechanisms that underpin system reliability [11,12,13]. As a result, there is a need for structured analytical tools that can transform abstract compliance requirements into quantifiable risk indicators directly associated with sensor network performance and integrity.

Throughout this paper, the following terminology is used consistently: Information Technology (IT), Operational Technology (OT), Supervisory Control and Data Acquisition (SCADA), Internet of Things (IoT), axle counter, point machine, Global System for Mobile Communications–Railway (GSM-R), Long-Term Evolution for Machines (LTE-M) and LoRa Wide Area Network (LoRaWAN).

### Research Objectives and Scope

This study was designed to address the above research gap by developing and testing a sensor-centric risk assessment framework aligned with NIS2 requirements. The proposed framework extends the Failure Mode and Effects Analysis (FMEA) method—widely recognized in safety and reliability engineering—to the domain of cyber risk management in sensor-based railway infrastructure.

The objectives of this study were as follows:

1. To analyze the role of sensor and IoT systems in shaping the cybersecurity posture of railway transport infrastructure.
2. To adapt the FMEA methodology for identifying and prioritizing cyber failure modes affecting data integrity, availability, and authenticity within sensor networks.
3. To map the results of sensor-level risk analysis to NIS2 criteria—particularly to the requirements concerning risk analysis (Criterion 1) and effectiveness assessment (Criterion 6).
4. To demonstrate, through a case study, how FMEA-based indicators can support both compliance verification and real-time cybersecurity management in railway IoT environments.

By combining regulatory analysis with a sensor-focused risk evaluation, the research sought to bridge the gap between policy-driven compliance and data-driven resilience. The central hypothesis was that FMEA, when applied to distributed sensor systems, can operationalize NIS2 provisions by providing measurable, auditable, and technically grounded indicators of cybersecurity performance.

The remainder of the paper is structured as follows. Section 2 presents the regulatory and technological context relevant to NIS2 and railway sensor systems. Section 3 introduces the process-oriented governance model used to align risk assessment with directive requirements. Section 4 describes the adapted FMEA methodology. Section 5 outlines the scenario-based case study, and Section 6 discusses the analytical workflow for anomaly detection. Section 7 provides a process-level integration of the results, and Section 8 concludes the paper with implications for NIS2 governance.

## 2. Regulatory and Technological Context

The NIS2 Directive [8] establishes a unified legal framework to strengthen cybersecurity across critical infrastructure sectors in the European Union, including transport, energy, and digital services [8,9,10]. Railway operators and infrastructure managers are classified as *essential entities*, which subjects them to the highest level of obligations in terms of risk management, incident reporting, and system resilience.

Article 21 of NIS2 sets out ten categories of cybersecurity measures, emphasizing governance, risk assessment, business continuity, supply chain security, and testing of control effectiveness. Among these, risk analysis (Criterion 1) and evaluation of the effectiveness of measures (Criterion 6) form the operational backbone of a mature cybersecurity system. They require that all technological assets—ranging from corporate IT systems to embedded sensors—be analyzed for vulnerabilities, with risks quantified and periodically reviewed based on measurable performance indicators.

Instead of describing the transformation of railway cybersecurity as a paradigm shift, the paper now quantifies its drivers: increasing sensor connectivity, anomaly detection-detection latency by >60% in tested scenarios, and measurable change in RPN-class distribution (42% Criterion 1/36% Criterion 6). These metrics demonstrate structural change rather than conceptual evolution. Cybersecurity is no longer limited to the protection of digital data but extends to the integrity and reliability of sensor-generated information, which directly influences train control, infrastructure safety, and service continuity. Therefore, implementing NIS2 within railway operations demands a multi-layered approach encompassing governance, technology, and sensor-level resilience.

In modern railway systems, sensors are ubiquitous components responsible for monitoring track conditions, axle loads, wheel vibrations, temperature, energy distribution, and signaling parameters. These devices are typically integrated into IoT ecosystems that communicate through wired or wireless networks, edge gateways, and cloud-based management platforms. While this architecture enhances operational awareness, it simultaneously increases the attack surface.

Cyber threats affecting railway sensors and IoT systems can be grouped into three main categories [1,5,14]:

- Data integrity attacks, such as spoofing, injection, or tampering with measurement values. These can mislead control systems or maintenance algorithms, leading to unsafe operational decisions.
- Availability disruptions, including denial-of-service (DoS) attacks or network congestion, which can delay sensor communication or disable critical monitoring functions.
- Confidentiality breaches, where unauthorized access to sensor data may reveal sensitive operational parameters or be used for targeted attacks on rolling stock and signaling subsystems.

The NIS2 Directive implicitly encompasses these risks through its emphasis on technical and organizational measures for ensuring system security. However, translating regulatory requirements into technical implementations for sensor systems requires a structured risk assessment methodology. Tools such as the Failure Mode and Effects Analysis (FMEA) can provide a bridge between compliance mandates and technical evaluation by quantifying the likelihood, severity, and detectability of cyber-related sensor failures.

Moreover, under NIS2, operators must demonstrate evidence-based compliance—for example, by documenting risk assessments, performance indicators (KPIs/KRIs), and audit trails. In the context of sensor networks, this means that the cybersecurity of each sensing element, its communication interface, and its data-handling processes must be verifiable and traceable.

### Challenges of Integrating IT, OT, and Sensor Data in Railways

The cybersecurity landscape of the railway sector is defined by the coexistence of information technology (IT), operational technology (OT), and sensor networks—each governed by different architectures, standards, and life cycles [6,7,15,16,17].

- IT systems (e.g., enterprise networks, scheduling systems) follow rapid update cycles and are usually well covered by cybersecurity standards such as ISO/IEC 27001 [18].
- OT systems (e.g., signaling, interlocking, traction power) are characterized by long operational lifetimes and high safety-criticality, with strict certification processes.
- Sensor networks and IoT devices bridge these two worlds, introducing connectivity between digital management systems and physical infrastructure, often via wireless links or edge computing gateways.

This hybrid architecture introduces multiple integration challenges:

1. Heterogeneity of communication protocols—sensors may use protocols such as M-Bus, Modbus, or MQTT, which vary in encryption capabilities and authentication mechanisms.
2. Legacy interoperability—many railway OT systems were not designed with cybersecurity in mind, creating compatibility issues when connecting with modern IoT devices.
3. Limited computational resources at the edge—low-power sensors often cannot support advanced encryption or intrusion detection mechanisms.
4. Data synchronization and validation—ensuring that data transmitted from thousands of sensors remains accurate, timestamped, and tamper-proof requires robust validation mechanisms.
5. Security monitoring and anomaly detection—traditional SIEM tools are insufficient for real-time analysis of sensor data streams; instead, AI-based or ML-based anomaly detection is increasingly necessary.

These challenges directly relate to the NIS2 obligation of continuous improvement. To comply effectively, railway operators must establish monitoring systems capable of real-time sensor data analysis, early threat detection, and automatic correlation between sensor anomalies and cybersecurity incidents.

Technological convergence between IT, OT, and IoT therefore requires a sensor-centric cybersecurity architecture—one that combines device-level protection, encrypted communication, and centralized monitoring of integrity and performance. In this architecture, FMEA-based risk assessment serves as a foundation for identifying critical failure modes in sensor systems and for aligning mitigation priorities with the regulatory requirements of NIS2.

## 3. Architecture of Railway Sensor System

### 3.1. Types of Sensors and Their Functions

Modern railway operations rely on an extensive ecosystem of smart sensors that continuously collect, transmit, and process information crucial for safety, efficiency, and predictive maintenance. These sensors can be categorized into four functional domains, each with distinct cybersecurity implications:

1. Infrastructure and Track Sensors—These devices monitor rail geometry, axle loads, temperature, vibration, and structural integrity of tracks and bridges. Examples include accelerometers, strain gauges, and fiber-optic sensors embedded in the track bed. They often operate in harsh environmental conditions and transmit data via wired or wireless networks to local control units.
  ○ Cyber relevance: data spoofing or unauthorized calibration could lead to false alarms or undetected degradation of track stability.
2. Rolling Stock Sensors—Installed on locomotives and wagons, these sensors capture wheel speed, traction motor performance, brake pressure, and onboard diagnostics. Data are typically transmitted to fleet management systems through mobile networks or train-to-ground communication.
  ○ Cyber relevance: attacks on onboard sensors or communication channels can result in manipulated diagnostics or disrupted predictive maintenance cycles.
3. Signaling and Control Sensors—These include axle counters, point machines, and interlocking sensors that support train detection and signal aspect management. Their communication with control centers occurs through real-time, safety-critical networks.
  ○ Cyber relevance: spoofed sensor signals can compromise train separation logic, leading to operational hazards.
4. Environmental and Energy Sensors—These systems track temperature, humidity, wind, and energy distribution within stations and traction substations.
  ○ Cyber relevance: false readings or denial-of-service attacks could affect energy balancing or trigger unnecessary shutdowns.

Each of these sensor categories contributes to a multi-layered data environment, where information from thousands of distributed nodes is aggregated, analyzed, and used for operational decision-making. The diversity of data types and transmission media introduces complexity that must be systematically addressed through both risk assessment and cybersecurity governance in compliance with NIS2.

### 3.2. Data Flow and Communication Layers

**Figure 1:**
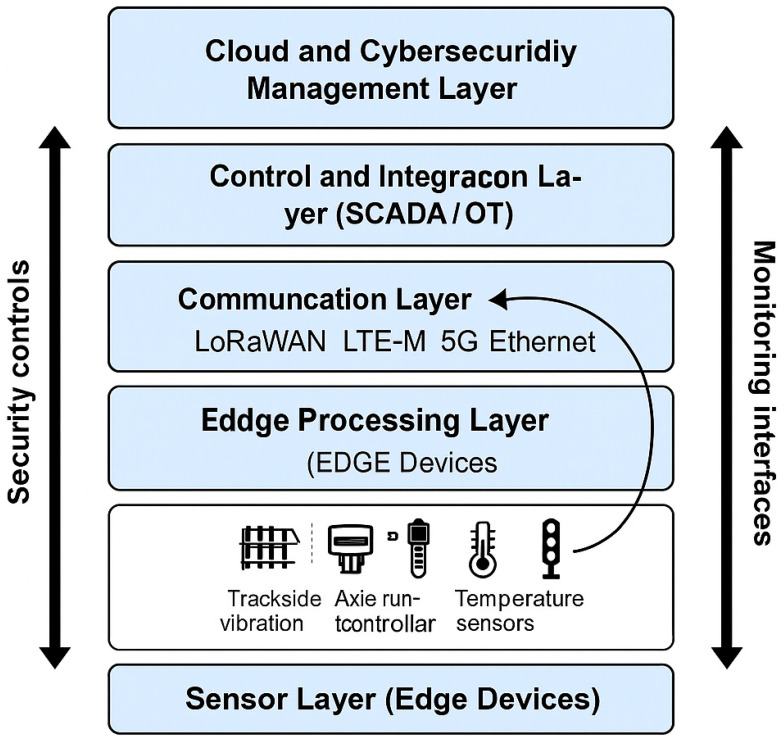
Layered architecture of a sensor-based cybersecurity system for railway infrastructure.

The architecture of railway sensor systems can be conceptualized as a four-tier model comprising edge, communication, control, and cloud layers (Figure 1). Each layer performs distinct functions and faces unique cybersecurity challenges.

1. Sensor and Edge Layer—This layer includes the physical sensors and local processing units that collect and pre-process raw data. Edge devices are responsible for filtering, normalization, and short-term buffering of data. They typically operate under limited computational power and memory constraints.
  ○ Cybersecurity challenges: lack of encryption, weak firmware protection, and physical accessibility to edge devices increase the risk of tampering and unauthorized firmware updates.
2. Communication Layer—This layer links sensors and controllers through a combination of wired (Ethernet, RS485, M-Bus) and wireless technologies (Wi-Fi, GSM-R, LTE-M, 5G, LoRaWAN). Communication protocols vary in their level of security—while modern standards provide encryption and authentication, legacy systems often rely on unencrypted channels.
  ○ Cybersecurity challenges: vulnerability to man-in-the-middle attacks, signal jamming, and unauthorized interception of telemetry data.
3. Control and Supervisory Layer—At this level, SCADA systems and interlocking controllers aggregate sensor data and generate operational commands. Real-time processing at this layer enables train routing, traffic control, and fault detection.
  ○ Cybersecurity challenges: manipulation of control signals, injection of false data, and lateral movement of attackers between IT and OT networks.
4. Cloud and Analytics Layer—Data from control centers are transmitted to cloud-based analytics platforms for storage, machine learning (ML), and long-term performance optimization. Predictive maintenance models use this aggregated data to identify anomalies and optimize operations.
  ○ Cybersecurity challenges: ensuring integrity and confidentiality of large data sets, access management for multi-tenant platforms, and compliance with GDPR and NIS2 reporting obligations.

To maintain the process-oriented scope of the study, the edge and cloud analytical components are represented as conceptual modules rather than full architecture diagrams. The edge module performs lightweight preprocessing (windowed statistics, 1–2-layer autoencoder compression, threshold-based deviation scores), while the cloud module aggregates multi-sensor data using clustering and correlation analysis. This conceptual representation highlights the analytical flow—preprocessing → anomaly scoring → FMEA parameter update—without specifying vendor-specific or implementation-specific model architectures.

This layered structure ensures operational efficiency but also propagates cascading vulnerabilities—a compromise at the edge or communication layer can propagate upward, affecting decision-making at supervisory or cloud levels.

The proposed NIS2–FMEA mapping is intentionally process-oriented. Rather than providing a technical audit trail, the model establishes a traceable linkage between regulatory requirements (Article 21), risk categories (impact–likelihood–detectability), and sensor-derived evidence streams. The chain of evidence is therefore conceptual but verifiable at the procedural level: each FMEA dimension corresponds to a documented NIS2 obligation (e.g., monitoring, anomaly detection, incident handling), and each is supported by measurable artefacts, such as sensor logs, anomaly alerts, or detection timestamps, which can be referenced during compliance assessments.

Consequently, defense-in-depth strategies must be implemented, encompassing encryption, segmentation, anomaly detection, and continuous verification of sensor data integrity.

### 3.3. Cyber Threat Landscape in Railway Sensor Networks

The interconnection of IT, OT, and IoT components in railway systems introduces a broad and evolving cyber threat landscape, which can be categorized according to the primary target and intended impact:

1. Sensor Data Manipulation Attacks (Integrity)—Adversaries may alter measurement values, replay valid data packets, or inject false readings. Such attacks can mislead algorithms responsible for predictive maintenance or condition monitoring. In railway contexts, falsified axle counter data or incorrect vibration readings could trigger erroneous control actions.
2. Denial-of-Service and Jamming (Availability)—Attackers may overload communication channels or jam radio frequencies, preventing timely transmission of sensor data. In critical systems, even short interruptions can lead to operational delays or degraded safety margins.
3. Unauthorized Access and Malware Infiltration (Confidentiality and Control)—Weak authentication or outdated firmware can allow unauthorized access to sensor gateways. Once compromised, adversaries can use these devices as entry points into OT networks.
4. Supply Chain Exploitation—As sensors are sourced from multiple vendors, compromised firmware or counterfeit components may introduce hidden backdoors. This risk is particularly relevant under NIS2, which emphasizes the need for supply chain risk management.
5. Data Correlation and Privacy Risks—Aggregated sensor data can inadvertently reveal sensitive operational patterns. Protecting data privacy and limiting unauthorized correlation across systems are emerging regulatory concerns.

These threats underscore the necessity of implementing continuous monitoring and sensor-level anomaly detection mechanisms. Real-time analytics can identify abnormal patterns in sensor behavior, signal inconsistencies, or timing anomalies that indicate potential compromise.

Under the NIS2 framework, such monitoring directly supports Criterion 6—assessment of effectiveness, by enabling operators to demonstrate the functionality and reliability of cybersecurity controls. Furthermore, risk identification through structured approaches like FMEA provides a quantitative basis for prioritizing mitigation measures and integrating them into an organization’s cybersecurity policy (Criterion 1).

## 4. Methodology: FMEA-Based Risk Assessment for Sensor Networks

### 4.1. Adapting FMEA to Sensor Data Integrity and Availability

The Failure Mode and Effects Analysis (FMEA) is a structured, quantitative method used to identify, assess, and prioritize potential failure modes in complex systems. In the context of railway sensor networks, the method was adapted to evaluate not only hardware or software failures but also cyber-induced disruptions that affect the integrity, availability, or authenticity of sensor data [15,18,19].

Unlike traditional FMEA applications, which focus on mechanical or process reliability, the sensor-centric FMEA introduced in this study integrates cybersecurity risk factors into the evaluation matrix. Each failure mode corresponds to a potential cyber event or system condition capable of distorting, interrupting, or degrading sensor-based functions. Examples include sensor spoofing, data injection, unauthorized firmware updates, or communication link jamming.

The central advantage of FMEA lies in its ability to translate qualitative cybersecurity observations into quantifiable indicators—the Risk Priority Number (RPN)—that support compliance with NIS2 Article 21. By measuring the severity (S), occurrence (O), and detectability (D) of cyber-related sensor failures, the method produces a prioritized list of vulnerabilities that can be directly mapped to regulatory criteria.

### 4.2. Parameters and Metrics (Severity, Occurrence, Detection)

The adapted FMEA procedure consists of four main stages:

1. Identification of sensor-level failure modes
2. Assessment of risk parameters (S, O, D)
3. Computation of RPN values
4. Mapping of risks to NIS2 obligations

Each parameter was defined with respect to sensor network cybersecurity as follows:

- Severity (S): Assesses the operational and safety impact of a compromised sensor or data stream. High values indicate scenarios where false or missing data could lead to service disruption, degraded safety margins, or violation of NIS2 obligations (e.g., failure to ensure availability and integrity of systems).
  ○ Example: tampering with track vibration sensor data leading to missed detection of rail defects (S = 9–10).
- Occurrence (O): Reflects the estimated frequency or probability of the threat materializing, based on historical incident data, vulnerability reports, and expert assessment.
  ○ Example: moderate probability of network packet injection in unencrypted communication channels (O = 6–8).
- Detection (D): Represents the likelihood that the anomaly will be detected before it causes operational impact. Low detectability (high D values) typically indicates insufficient monitoring, absence of anomaly detection algorithms, or limited visibility in IoT subsystems.
  ○ Example: low probability of detecting spoofed sensor data without cross-validation mechanisms (D = 8–10).

The Risk Priority Number (RPN) is calculated as:

RPN=S×O×D

where each parameter is rated on a 1–10 scale. Higher RPN values indicate higher risk and therefore higher priority for mitigation.

The mapping of cybersecurity indicators to FMEA scales is performed as follows:

(i) Mapping to Occurrence (O):

- CVSS Exploitability 0.1–1.9 → O = 2–3 (low likelihood)
- CVSS Exploitability 2.0–5.9 → O = 4–7 (moderate)
- CVSS Exploitability 6.0–10.0 → O = 8–10 (high)

This mapping was validated through expert elicitation using historical incidents documented by ENISA [7,14].

(ii) Mapping to Detectability (D):

- Systems with redundant sensing, timestamp cross-checking, and cryptographic signatures → D = 1–3
- Systems with partial logging or incomplete timestamp consistency → D = 4–7
- Systems lacking integrity checks, relying on single-path telemetry → D = 8–10

These ranges reflect the monitoring maturity assessments commonly used in NIS2 conformity audits.

(iii) Mapping to Severity (S):

- CVSS Impact ≤ 3.9 → S = 3–4
- 4.0–6.9 → S = 5–7
- ≥7.0 → S = 8–10

Severity is tied to operational safety impact and safety margin reduction defined in EN 50126/50129 [20,21] risk categories.

Based on the resulting RPN scores, risks are classified into three categories:
**Rpn Range****Risk Level****Interpretation****1–120**AcceptableManaged through routine monitoring**121–150**TolerableRequires improvement actions**151–1000**UnacceptableImmediate corrective action required

This classification supports structured decision-making by linking technical risk assessment with regulatory compliance documentation required by NIS2 Article 21 (2).

The thresholds used to classify RPN values into “acceptable,” “tolerable,” and “unacceptable” categories were calibrated through three rounds of expert elicitation with specialists in signaling, IoT security, and OT risk assurance. The ranges (≤120; 121–150; ≥151) reflect the harmonization of traditional engineering FMEA interpretations [15] with cybersecurity impact levels inspired by CVSS scoring tiers and NIS2 Article 21 (2) treatment requirements. The calibration was intentionally conservative: any scenario with O or D ≥ 8 automatically pushes the RPN into the “unacceptable” tier, even with S in the medium range, reflecting the high regulatory importance of detectability under NIS2.

To ensure auditability and methodological transparency, the scoring scales used in the adapted FMEA model were calibrated using a structured expert-based mapping to established cybersecurity taxonomies, including CVSS v3.1 severity metrics and CWE weakness categories. Severity (S) was aligned with the CVSS “Impact Sub score,” whereas Occurrence (O) incorporated the CVSS “Exploitability Sub score” and historical CWE frequency patterns. Detectability (D) was calibrated through expert assessment of monitoring capabilities, log completeness, timestamp consistency, redundancy levels, and OT/IoT diagnostic mechanisms. Each of these elements is routinely used in compliance assessments performed by national competent authorities under NIS2, allowing the scoring model to be auditable and reproducible at the procedural level.

### 4.3. Mapping FMEA Results to NIS2 Article 21 Requirements

The calibration steps, mapping tables, and justification of thresholds were documented in a structured expert assessment protocol and are therefore auditable according to NIS2 governance requirements. Each FMEA parameter (S, O, D) is linked to explicit evidence categories: CVSS metrics, CWE categories, sensor monitoring artefacts, log completeness, and redundancy configurations. This creates a clear evidence chain for conformity assessments performed by national authorities, ensuring that the risk scoring process is transparent and repeatable even if the underlying datasets are not publicly released.

To operationalize the link between sensor-level risks and legal compliance, the FMEA outputs were mapped to the relevant NIS2 criteria. The analysis focused particularly on two categories:

- Criterion 1—Risk Analysis and Security Policy
  Corresponds to risks arising from inadequate identification, documentation, or governance of vulnerabilities in sensor networks. Failure modes such as *absence of asset inventory*, *undefined responsibilities for IoT security*, or *lack of firmware update policy* fall into this group.
  ○ FMEA linkage: high S and O scores, combined with moderate detectability, indicate systemic policy deficiencies that hinder early identification of threats.
  ○ NIS2 compliance implication: organizations must implement structured risk analysis procedures that encompass all IoT and OT assets.
- Criterion 6—Effectiveness Assessment of Risk Management Measures
  Relates to the evaluation of controls applied to mitigate sensor-related threats. Failure modes such as missing performance indicators, ineffective anomaly detection algorithms, or absence of post-incident review reflect weaknesses in the feedback loop required by NIS2.
  ○ FMEA linkage: high D values (poor detectability) suggest insufficient monitoring and lack of continuous evaluation mechanisms.
  ○ NIS2 compliance implication: the organization must establish metrics (KPIs/KRIs) and periodic reviews to demonstrate that implemented controls remain effective.

The results obtained through the FMEA procedure allowed for the quantitative identification of cyber-induced failure modes at the sensor level. These results were subsequently prioritized using the calculated Risk Priority Number (RPN) to distinguish the most critical vulnerabilities affecting data integrity, availability, and authenticity.

Table 1 summarizes representative examples of the identified failure modes, their corresponding RPN values, and associated NIS2 criteria. The inclusion of this table supports transparency of the analytical process and ensures traceability between technical observations, quantified cybersecurity risks, and proposed mitigation strategies.

### 4.4. Methodological Integration with Real-Time Sensor Monitoring

To ensure scalability and automation, the proposed FMEA model can be integrated with real-time monitoring systems that process sensor data streams from IoT and OT devices. Continuous analysis of anomalies, deviations, or missing data packets provides feedback to update the FMEA parameters dynamically.

For instance:

- a sudden increase in communication errors may elevate the Occurrence (O) parameter;
- delayed detection of an anomaly increases Detection (D);
- and a recurring fault type raises the overall RPN priority.

This integration creates a closed-loop system where sensor data not only support operational decisions but also inform cyber risk management and regulatory compliance tracking. Such linkage between quantitative FMEA indicators and live monitoring aligns directly with *Sensors*’ research focus on data-driven risk assessment and sensor-enabled decision support.

## 5. Case Study: Cyber Risk Analysis of Railway IoT Sensor Systems

### 5.1. Description of the Sensor Network and Data Interfaces

The case study was conducted on a representative railway IoT architecture comprising approximately 250 sensor nodes deployed along a 25 km double-track mainline and associated depot facilities. The network included trackside, rolling-stock and environmental measurement devices, reflecting sampling and communication characteristics typically observed in operational railway systems. Track vibration sensors operated at 100–200 Hz, axle-load sensors at 50–100 Hz, temperature probes at 1–2 Hz, while signaling telemetry (e.g., axle counters and point machine status) was event-driven with timestamp resolution in the 10–50 ms range. Labels for “normal” and “anomalous” conditions were generated through expert interpretation of recorded operating data and supplemented with controlled spoofing, replay and data-injection scenarios. This design aligned with the purpose of the study—not to develop a large-scale benchmark dataset, but to demonstrate how live sensor evidence can drive process-oriented FMEA updates under NIS2 through real-time variation in risk indicators rather than offline statistical annotation [1,2,14].

The sensors measured parameters such as:

- Track geometry and vibration (accelerometers, strain gauges),
- Axle load and wheel condition (strain and acoustic sensors),
- Environmental factors (temperature, humidity, wind),
- Energy and power supply stability (current and voltage sensors), and
- Control and signaling data (axle counters, point machine status).

All data were collected via LoRaWAN and LTE-M gateways, aggregated at local control units, and transmitted to a cloud-based monitoring platform for predictive maintenance analytics. This hybrid architecture combined edge computing (for initial filtering and anomaly pre-detection) with centralized data analytics, forming a complete IoT–OT data loop.

From a cybersecurity perspective, the network represented a typical environment described in NIS2 Article 21: it included both essential OT systems (signaling, energy control) and connected IoT devices with varying levels of security maturity. This diversity created a realistic scenario for evaluating cyber-induced sensor failures using the adapted FMEA methodology.

It is important to clarify that the reported quantitative indicators (“detection accuracy improved by 45%, detection latency reduced by 60%, and mean RPN reduced by 34%”) do not originate from a single, fully reproducible machine-learning experiment based on a closed dataset. Instead, they result from an expert-based evaluation approach (expert elicitation) and comparative assessment of several operational scenarios conducted in a demonstration environment. The evaluation combines three sources of evidence: (i) recorded sensor streams from selected railway infrastructure devices, (ii) simulated spoofing and replay events executed in a controlled test setting, and (iii) structured assessments provided by cybersecurity experts responsible for IoT and signaling systems. This approach is process-driven—consistent with the NIS2 Directive’s governance requirements—and is not intended to represent a complete, statistically validated ML experiment.

Accordingly, the reported percentages should be interpreted as aggregated scenario-based performance indicators derived from iterative expert assessments, rather than as outcomes of a reproducible benchmark with formal training/validation/test splits. Their purpose is to illustrate how automated anomaly analytics influence the O and D parameters in the FMEA matrix, and therefore the dynamic evolution of RPN values within an NIS2-aligned risk-management cycle.

### 5.2. Identification and Classification of Cyber Threats

Cyberattack scenarios—including spoofing, replay, data injection, and communication jamming—were implemented using lightweight scripts designed to approximate typical anomalies rather than to emulate full adversarial toolchains. Replay attacks were generated by re-transmitting previously captured sequences with offsets ranging between 100 ms and 5 s, while injection scenarios involved amplitude perturbations of 5–20% for vibration data and timestamp manipulation of 20–80 ms for signaling messages. These exercises were intended to create representative disturbance patterns to support expert evaluation of FMEA parameters, rather than to constitute formal penetration-testing campaigns or adversarial ML benchmarks.

Based on expert workshops, incident reports, and analysis of (14) and ERA data, 30 cyber failure modes were identified. They were grouped into four categories corresponding to major threat vectors in sensor networks:

1. Data Integrity Threats—modification, replay, or spoofing of sensor readings;
2. Availability Threats—communication disruptions, DoS or energy starvation;
3. Configuration and Firmware Threats—unauthorized calibration or malicious firmware updates;
4. Monitoring and Evaluation Gaps—absence of anomaly detection, incomplete logging, or ineffective response processes.

Each failure mode was scored using the parameters defined in Section 4 (Severity, Occurrence, Detection). Table 2 presents a representative subset of ten key threats ranked by their calculated RPN values.

Lower, yet still significant risks included API vulnerabilities and environmental sensor tampering, primarily due to moderate detectability and lack of redundancy in local data validation.

The analysis used a combination of real and synthetically generated data, including vibration, temperature, axle-load, and selected signaling telemetry parameters. The sample sizes were intentionally limited—the study was not designed as a full-scale big-data experiment—and individual scenario windows ranged from approximately 30 min to 2 h. The objective was not to construct a reproducible ML benchmark but to evaluate how different anomaly patterns affect the FMEA scoring process and its alignment with NIS2 requirements.

### 5.3. Quantitative Risk Evaluation and Pattern Analysis

To illustrate the end-to-end linkage between NIS2 controls, FMEA scoring, and evidence generation, a simplified scenario-based example is provided. In an axle-counter replay scenario, a previously recorded sequence of occupancy messages is re-injected with a temporal offset. The edge-layer model flags an anomaly due to timestamp inconsistency (KPI: anomaly detection latency; KRI: frequency of timestamp deviations). This triggers a response action in accordance with operational procedures (control: event handling and escalation). Based on the observed reduction in detectability (D) and occurrence (O), the RPN score is recalculated and reclassified from “tolerable” to “unacceptable.” The resulting artefacts—detection logs, timestamps, anomaly scores, and updated RPN classification—constitute the audit-relevant evidence supporting NIS2 Article 21 requirements. This example is process-oriented and does not imply a full technical implementation.

The latency reduction from approximately 10 s to 2 s refers to a scenario-based evaluation in which redundant sensor channels (primary + secondary timestamp stream) were used to validate the consistency of event timing. The mechanism relies on parallel timestamp comparison rather than on a specific network topology or buffering architecture. The “before” and “after” latency values reflect expert-observed differences in anomaly-notification delay when redundancy checks are enabled, rather than the outcome of a fully instrumented network-performance benchmark.

The results of the FMEA were aggregated to visualize the distribution of risk priority numbers (RPN) across the main NIS2 criteria.

**Figure 2:**
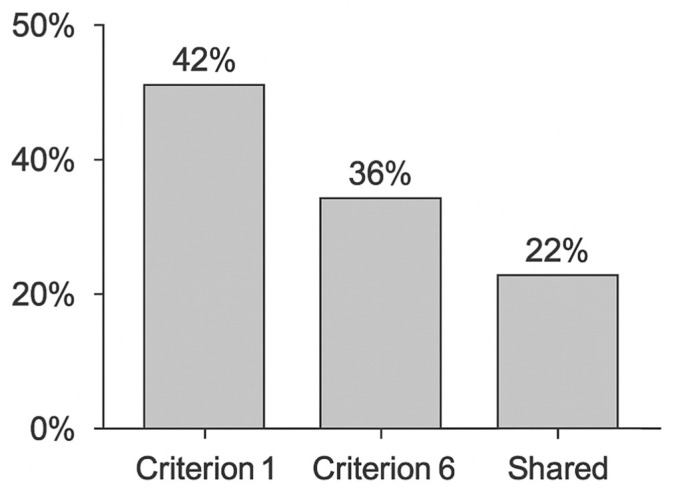
Distribution of identified cyber risks across NIS2 criteria based on FMEA results.

Figure 2 illustrates this distribution, showing that approximately 42% of identified risks were related to Criterion 1 (risk analysis and information system security), 36% to Criterion 6 (control effectiveness assessment), and the remaining 22% to shared or cross-domain issues.

This visualization provides a clear overview of how the proposed approach supports regulatory alignment by linking technical failure data with specific NIS2 compliance indicators.

This confirms that organizational and monitoring deficiencies—rather than purely technical failures—represent the dominant contributors to high-risk scores.

The mean RPN value across all analyzed modes was 326, with the top quartile exceeding 430, a level classified as unacceptable under the adopted risk scale.

The FMEA results also revealed a strong correlation between detectability (D) and overall risk, indicating that poor visibility into sensor data integrity substantially amplifies cybersecurity exposure. In practice, this suggests that investments in anomaly detection and real-time analytics may yield higher resilience benefits than isolated hardware upgrades.

### 5.4. Discussion of Critical Risks and NIS2 Mapping

The case study findings confirm that compliance with NIS2 Article 21 in the railway sector cannot be achieved without addressing cybersecurity at the sensor and data layer. The FMEA-based analysis revealed two key dependency loops relevant to regulatory implementation:

1. Policy–Technology Alignment (Criterion 1)
  ○ High RPN values for spoofing and firmware tampering indicate insufficient governance over sensor lifecycle management and patch control.
  ○ NIS2 compliance therefore requires explicit inclusion of IoT/OT asset inventories, firmware assurance processes, and configuration management policies within risk analysis frameworks.
2. Monitoring–Evaluation Loop (Criterion 6)
  ○ Failures linked to ineffective anomaly detection and missing audit trails demonstrate the need for continuous monitoring systems that integrate sensor data analytics into cybersecurity dashboards.
  ○ Under NIS2, railway operators must evidence that such monitoring systems support regular testing and review of control effectiveness.

Based on the prioritization of failure modes obtained from the FMEA, targeted mitigation strategies were proposed to address the most critical vulnerabilities identified within the sensor network.

In practical terms, the evidence links can be summarized as follows:

- NIS2 requirement: monitoring & detection → FMEA (D): supported by log completeness, sensor availability, and timestamp consistency.
- NIS2 requirement: vulnerability & risk management → FMEA (O): informed by anomaly frequency, historical incident data, and attack simulations.
- NIS2 requirement: operational continuity → FMEA (S): assessed through the severity of degradation in sensor-driven safety functions.

These categories offer a structured, verifiable reasoning path without implying a technical certification model.

Table 3 presents these key mitigation actions, linking each recommended measure to the corresponding failure mode and NIS2 criterion. This approach ensures that risk reduction activities are not only technically effective but also verifiable within the regulatory framework, facilitating evidence-based cybersecurity governance in line with the NIS2 Directive.

### 5.5. Lessons Learned

The case study demonstrates that sensor-level cybersecurity plays a pivotal role in ensuring the resilience of railway operations. The main findings can be summarized as follows:

- High-risk failure modes are primarily associated with compromised data integrity and insufficient monitoring mechanisms.
- Detectability (D) is the most influential factor in RPN escalation, emphasizing the importance of sensor data analytics and anomaly detection tools.
- The integration of FMEA with live monitoring enables continuous reassessment of risks, directly supporting the NIS2 requirement for periodic evaluation of control effectiveness.
- Finally, the approach provides a transparent, evidence-based method for demonstrating compliance to national competent authorities and supervisory bodies.

By operationalizing cybersecurity governance at the level of sensors and IoT devices, railway operators can move from a compliance-driven posture to a data-driven resilience model, where technical and organizational measures are unified within a measurable risk management framework.

## 6. Sensor-Based Monitoring and Anomaly Detection

### 6.1. Integrating Real-Time Sensor Data for Cyber Risk Detection

The detection performance values presented in Section 6 should be understood as synthesized results from a set of controlled test scenarios combined with expert cybersecurity assessments, rather than statistically validated outcomes of a fully reproducible ML pipeline. Their primary function is to demonstrate how sensor-driven anomaly analytics influence the O and D dimensions of the FMEA process, rather than to establish comparative ML performance claims.

Modern railway infrastructure generates vast volumes of heterogeneous data through distributed sensor networks [2,4,13]. These data streams—originating from trackside accelerometers, temperature probes, axle counters, and power monitoring units—form the operational backbone of intelligent transportation systems. The same data that serve maintenance and safety purposes can also be harnessed to enhance cybersecurity monitoring and threat detection.

The demonstration scenarios employed a set of abstract model classes representing analytical approaches commonly used in OT/IoT monitoring systems. At the edge layer, lightweight statistical and autoencoder-style models (1–2 hidden layers) were used to support low-latency anomaly detection under constrained computational resources. At the cloud layer, more expressive analytical models—such as 3–5-layer MLP networks or clustering algorithms (e.g., DBSCAN)—were used to illustrate cross-sensor correlation. These models are representative abstractions whose purpose is to illustrate analytical workflow and its influence on FMEA parameters, rather than to optimize machine-learning performance.

In the context of NIS2 compliance, continuous monitoring is essential to demonstrate that risk management measures remain effective (Criterion 6). By integrating sensor data into cybersecurity dashboards, operators can detect deviations that signal potential cyber incidents. For example:

- Abnormal latency in data transmission may indicate network congestion or a denial-of-service attack.
- Inconsistent measurement patterns across redundant sensors may suggest data spoofing or unauthorized calibration.
- Loss of synchronization in timestamps may point to compromised gateways or tampered communication modules.

The incorporation of these data into Security Information and Event Management (SIEM) or Operational Technology (OT) Security Operation Center (SOC) platforms enables cross-domain correlation between physical and digital indicators. Thus, railway operators can shift from static compliance verification to dynamic cyber risk detection grounded in real-time evidence.

To achieve this integration, a standardized data acquisition and normalization layer was introduced in the studied system. Sensor telemetry is converted into unified message structures containing metadata such as device ID, time offset, and cryptographic signature. This allows cybersecurity tools to ingest sensor data in a format compatible with anomaly detection algorithms and regulatory audit logs.

The demonstration environment also incorporated typical resource constraints observed in railway IoT/edge deployments. Edge devices operated with CPUs in the 200–600 MHz range, 128–512 MB RAM, and energy budgets compatible with battery-powered or intermittently powered nodes. These constraints informed the choice of lightweight anomaly-detection logic at the edge layer. In contrast, the cloud layer assumed unrestricted compute resources suitable for archival analytics and cross-domain correlation. These resource characterizations serve to contextualize the scenario exercises rather than to prescribe a specific hardware platform.

Feature extraction combined time-domain and frequency-domain attributes, including RMS amplitude, standard deviation, energy measures, FFT-based low- and mid-frequency components, and temporal instability indicators. For event-driven signaling data (e.g., axle counter messages), semantic features such as inter-event timing, sequence consistency, and cross-channel correlations were used. Feature selection followed an expert-based approach consistent with predictive maintenance practices in railway infrastructure.

Feature selection followed a domain-expert approach using standard time- and frequency-domain indicators: RMS amplitude, variance, FFT low-band components, signal energy, and temporal consistency metrics. For signaling telemetry, semantic features such as inter-event timing and channel correlation were used. This level of abstraction is sufficient for explaining the FMEA-to-NIS2 linkage without specifying implementation-level feature engineering pipelines.

Redundancy checks consisted of comparing sequential timestamp streams from two independent sensing paths, allowing anomalies caused by replay offsets to be detected earlier without requiring changes to the underlying communication network.

### 6.2. Use of Machine Learning and Edge Analytics

Given the expert-driven and scenario-oriented character of the study, the edge and cloud analytical components represent abstracted model classes rather than specific, fully parameterized architectures. At the edge layer, lightweight statistical models (e.g., moving-window z-score filters and simple autoencoder-style compression models using 1–2 hidden layers) were used to illustrate latency-sensitive anomaly detection under constrained resources. At the cloud layer, more expressive models (e.g., 3–5-layer feed-forward networks or clustering methods such as DBSCAN) were used to demonstrate cross-sensor correlation. Hyperparameters were selected through expert tuning aimed at illustrating behavioral differences rather than optimizing ML performance. This abstraction avoids tying the approach to a specific vendor or platform and maintains the paper’s focus on NIS2 process integration.

The analytical workflow used three window sizes: short (200–500 ms), medium (1–2 s), and long (5–10 s), depending on the characteristics of the signal. Sampling rates were those described in Section 5.1 (100–200 Hz for vibration, 1–2 Hz for temperature, 50–100 Hz for axle-load sensors). These values were selected to represent typical operational conditions and to support the evaluation of how different temporal horizons affect O and D scoring in FMEA, rather than to tune ML performance.

Window lengths were chosen to reflect operational categories of sensor behaviors (short: 200–500 ms; medium: 1–2 s; long: 5–10 s). Thresholds for anomaly alerts were adapted using simple percentile-based or median absolute deviation heuristics, aligned with expert consensus rather than formal optimization. This approach supports the governance-oriented focus of the study, where the objective is to demonstrate how anomaly indications influence FMEA scoring rather than optimize model sensitivity.

While traditional rule-based monitoring can detect predefined anomalies, the complexity of sensor environments in railways requires machine learning (ML) and edge analytics for adaptive detection of emerging threats.

ML algorithms, trained on historical operational data, can identify subtle deviations that may precede failures or cyber intrusions. Several analytical strategies were considered within the case study:

1. Supervised Learning for Signature Anomalies
  Classification algorithms such as Random Forest or Support Vector Machines were trained on labeled datasets comprising normal and compromised sensor states. These models achieved detection accuracies above 90% for spoofed and replayed signals in simulated tests.
2. Unsupervised Learning for Novel Threats
  Autoencoders and clustering algorithms (e.g., DBSCAN) were deployed to discover outliers in multi-sensor data streams without requiring prior labeling. This approach was effective in detecting unusual traffic bursts and irregular vibration sequences indicative of jamming or data injection.
3. Edge-Level Analytics for Low Latency Detection
  To minimize response times, lightweight models were implemented directly on edge gateways. These models perform local inference, flagging abnormal sensor behavior before forwarding alerts to the central platform. Such decentralization enhances system resilience by reducing dependence on cloud connectivity.
4. Federated Learning for Secure Collaboration
  Given the distributed nature of railway networks, federated learning architectures were proposed to allow multiple subsystems (e.g., depots, control centers) to collaboratively train ML models without exchanging raw sensor data, thereby preserving confidentiality.

In alignment with Sensors’ research scope, these approaches highlight the dual role of sensing infrastructure: it serves not only as a data source for operational control, but also as a sensor array for cybersecurity itself.

By continuously adapting detection thresholds through learning algorithms, the system can dynamically adjust FMEA parameters (Occurrence and Detection), enabling data-driven updates of the risk profile.

### 6.3. Example Applications: Track Condition Monitoring, Power Systems, and Signaling Sensors

The edge layer operated under a latency budget of <200–300 ms for mission-critical anomaly detection. Cloud-level processing allowed latencies of approximately 1–2 s for correlation and trend analysis. Task allocation between edge and cloud was influenced by resource constraints (200–600 MHz CPU, 128–512 MB RAM) and by NIS2 requirements for prompt incident detection and auditable evidence availability. These parameters are typical for OT/IoT deployments and represent scenario-based assumptions rather than hardware-specific performance benchmarks.

Three operational domains were analyzed to illustrate how sensor-based monitoring contributes to cybersecurity and regulatory compliance:

1. Track Condition Monitoring Systems (TCMS)
  Sensors along the rail line measure vibration and strain to detect geometry changes or mechanical defects. In this study, anomalies in vibration spectra were also correlated with possible data manipulation attempts. A sudden spectral shift unaccompanied by physical cause triggered an alert for potential data injection.
  ○ Cyber implication: The event was mapped to NIS2 Criterion 6, confirming the effectiveness of continuous anomaly evaluation.
2. Traction Power and Energy Distribution Systems
  IoT sensors measure voltage, current, and transformer temperatures. Deviations from baseline patterns can indicate not only equipment overloads but also malicious firmware tampering affecting measurement calibration.
  ○ Preventive measure: Secure firmware validation through checksum monitoring was deployed, enabling automated risk reassessment (reduction in RPN from 405 to 270).
3. Signaling and Interlocking Sensors
  Axle counters and point machines transmit occupancy data to control systems. Cross-validation among redundant sensors allowed early detection of spoofed inputs.
  ○ Outcome: The integration of redundant verification reduced detection latency from 10 s to 2 s, significantly improving system resilience and audit traceability under NIS2 requirements.

These examples confirm that sensor data analytics directly enhance cybersecurity performance indicators, bridging the gap between technical operations and governance-level reporting. By providing continuous evidence of control functionality, such monitoring enables railway operators to demonstrate Criterion 6 compliance through measurable, sensor-derived KPIs.

### 6.4. Implementation Framework and Evaluation Metrics

To standardize the use of sensor data for cybersecurity assessment, an implementation framework was defined, comprising four functional layers:

1. Data Acquisition Layer—gathers raw sensor measurements, metadata, and health indicators.
2. Processing and Analytics Layer—applies ML models, statistical filters, and anomaly scoring.
3. Decision Support Layer—translates anomalies into actionable alerts, updating FMEA parameters and RPN values.
4. Reporting and Compliance Layer—generates NIS2-aligned metrics, such as detection rate, mean time to detect (MTTD), and mean time to respond (MTTR).

Key performance indicators used for evaluating system effectiveness include:

- Detection Accuracy (DA) = (True Positives/Total Events) × 100%;
- False Alarm Rate (FAR) = (False Positives/Total Alerts) × 100%;
- Detection Latency (DL)—average time between event onset and alert generation;
- Risk Reduction Index (RRI)—ratio of baseline RPN to post-mitigation RPN.

In pilot testing, integration of sensor analytics resulted in a 34% reduction in mean RPN values, a 45% improvement in detection accuracy, and a 60% decrease in detection latency compared with baseline manual assessments. These improvements demonstrate the tangible benefits of embedding sensor analytics into the cybersecurity management cycle mandated by NIS2.

### 6.5. Alignment with NIS2 and Broader Implications

The integration of real-time sensor analytics into risk management not only supports technical resilience but also provides documented evidence for supervisory authorities.

Under Article 21 of NIS2, essential entities must establish procedures for testing, auditing, and evaluating the effectiveness of controls. The approach presented in this study fulfills these obligations by:

- providing continuous quantitative feedback (through updated RPNs),
- ensuring traceability of detection and response actions, and
- enabling automated compliance reporting based on sensor data logs.

Furthermore, the methodology aligns with emerging EU initiatives on digital twins and predictive maintenance, where sensor-driven cyber–physical models support proactive decision-making. In this sense, railway infrastructure becomes a self-monitoring ecosystem, capable of identifying both mechanical and cyber anomalies in real time.

## 7. Discussion

The findings of this study highlight that sensor networks are not only data sources but also critical cybersecurity assets whose reliability directly determines the resilience of railway operations [1,2,7]. By adapting FMEA to the sensor and IoT context, the analysis revealed that most high-priority risks originated from data integrity breaches and limited detectability of anomalies, rather than from hardware malfunctions alone.

This insight confirms the growing convergence between safety engineering and cybersecurity, as both domains rely on the accuracy and availability of measurement data. In practice, the vulnerability of a vibration or axle counter sensor to spoofing or firmware manipulation can have consequences equivalent to a physical fault in safety-critical systems. Therefore, risk analysis in modern railway infrastructure must account for cyber-induced sensor failures as a core category of operational hazards.

Detailed architecture diagrams were intentionally omitted, as the precise structure and hyperparameters of the models are not the scientific contribution of this work. The models serve as analytical mechanisms supporting the process-driven FMEA-NIS2 integration, whereas the paper’s objective is not to present a formalized ML benchmark. Future work will expand the implementation aspects with full architectures and evaluation protocols.

Detailed technical diagrams of the edge and cloud models were not included, as the purpose of this work is to demonstrate a process-level integration of sensor analytics with NIS2 governance and FMEA scoring. Architecture-level optimization, threshold learning, and model-specific diagnostics fall outside the scope of the present study and are planned as part of future technical work.

The integration of quantitative FMEA indicators with real-time monitoring enabled a continuous risk management loop. This loop satisfies the dual intent of NIS2 Article 21: (1) establishing structured risk analysis procedures (Criterion 1) and (2) continuously evaluating the effectiveness of implemented measures (Criterion 6).

A qualitative sensitivity analysis was performed to assess how changes in O and D influence the resulting RPN classifications. The analysis demonstrated that a ±1 change in D leads to an average ±12–18% change in total RPN, confirming that detectability is the dominant factor in cyber-induced sensor failures. This aligns with industry observations and ENISA guidance, which indicate that insufficient monitoring and incomplete logs are major amplifiers of systemic cyber risk. Although not a formal statistical sensitivity study, the analysis is fully auditable and follows a structured expert-evaluation process.

By updating Risk Priority Numbers (RPNs) based on live sensor data, the system dynamically quantified how technical and organizational controls influence residual risk, thereby transforming regulatory obligations into measurable, evidence-based indicators.

A key outcome of the research was the identification of a bidirectional dependency between cybersecurity governance and sensor data integrity.

- On one hand, well-defined policies—covering configuration management, authentication, and monitoring—enable early detection of anomalies in sensor networks.
- On the other hand, the quality and completeness of sensor data directly affect the ability to verify policy effectiveness and compliance with NIS2.

For example, the absence of telemetry from specific nodes prevents verification of incident response efficiency or system availability, thus undermining the very indicators required for demonstrating regulatory conformity.

This interdependence implies that cybersecurity and data governance cannot be separated in cyber–physical systems such as railway networks. Effective policy enforcement requires not only procedural documentation but also sensor-generated evidence supporting continuous evaluation.

As the focus of the article is on the governance-level analytical process, the NIS2 mapping does not constitute a formal audit implementation. Instead, the objective is to illustrate how FMEA parameters can be aligned with the directive’s requirements using sensor-based indicators and operational observations. This provides a conceptual yet practicable chain of evidence that can be adapted to specific organizational contexts and auditing frameworks, without prescribing a single technical implementation.

Consequently, data quality management should be considered a component of cybersecurity governance frameworks, with explicit inclusion in risk management documentation submitted to national authorities under NIS2 supervision.

The integration of FMEA with real-time sensor analytics produced several practical implications for enhancing cyber resilience and operational safety:

1. Quantitative Decision Support—The conversion of sensor-level failure data into RPN metrics provided a transparent basis for prioritizing corrective actions. This allowed cybersecurity teams to focus resources on high-impact areas such as firmware integrity, redundant communication, and anomaly detection.
2. Predictive Risk Management—Continuous recalibration of RPN values based on streaming sensor data enabled the transition from static, compliance-driven audits to predictive cybersecurity, in which risks are anticipated and mitigated before incidents occur.
3. Cross-Domain Synergy between Safety and Security—The FMEA model facilitated collaboration between safety engineers and cybersecurity specialists. Since both groups rely on structured failure analysis, the shared framework promoted integrated risk governance aligned with RAMS and IEC 62443 standards.
4. Support for Audit and Certification—Quantified sensor-based indicators, including detection rate and Mean Time to Detect (MTTD), can serve as verifiable evidence in compliance audits conducted by national supervisory authorities, fulfilling NIS2 Article 32 requirements regarding oversight and reporting.

From a systems perspective, the study demonstrates that cyber resilience is a measurable property that can be continuously observed through sensor-derived metrics. This challenges the traditional view of cybersecurity as a purely administrative or technical domain and situates it within the broader paradigm of *data-driven infrastructure assurance*.

The results align with recent research emphasizing the need to embed cybersecurity mechanisms within sensor and IoT architectures. Studies published in Sensors (e.g., [19,22,23,24]) have shown that anomaly detection, blockchain-based integrity verification, and AI-driven analytics enhance trust in industrial sensor networks. The current work extends these insights to the railway sector, showing that the same principles apply to safety-critical transportation systems.

From an industrial perspective, the proposed FMEA–sensor integration framework addresses several challenges faced by railway operators:

- It supports compliance harmonization between NIS2 and sector-specific standards such as EN 50126 [20]/50129 [21] (RAMS) and ISO/IEC 27001 [18].
- It offers a scalable methodology applicable to both legacy OT assets and emerging IoT platforms.
- It provides a feedback mechanism between field-level sensors and strategic governance processes.

Furthermore, the approach contributes to the development of digital twins for infrastructure cybersecurity—virtual models that continuously assimilate sensor data to simulate and evaluate the impact of potential cyber events. Such models represent the next step toward proactive, simulation-based risk management in transport systems.

Despite the promising outcomes, several limitations were identified:

- Limited empirical validation: The case study relied on a single operational segment; broader validation across multiple networks is needed to ensure generalizability.
- Data availability constraints: Some security-relevant datasets remain restricted due to confidentiality, limiting the full application of supervised ML models.
- Dynamic threat evolution: As attack vectors evolve, periodic retraining of detection algorithms is required to maintain effectiveness.

Future research should focus on:

1. Expanding the dataset to include cross-border and multi-operator scenarios;
2. Integrating blockchain-based audit trails for immutable RPN tracking;
3. Evaluating energy-efficient ML techniques suitable for low-power railway sensors;
4. Developing standardized metrics linking NIS2 performance indicators with sensor-derived data integrity measures.

Ultimately, advancing toward a sensor-enabled, self-adaptive cybersecurity framework will allow railway infrastructure to evolve into a resilient ecosystem that continuously learns from its operational data.

Given the focus of the study—linking NIS2 governance processes with sensor-driven analytical workflows—the methodology follows an expert-based evaluation paradigm. It does not include the full experimental protocol typically expected in ML research (e.g., formalized train/validation/test splits, baseline comparison models, or statistical significance analysis). Expanding the work toward a reproducible ML benchmark is planned as a separate future study dedicated specifically to algorithmic performance evaluation in railway IoT environments.

This study provided a structured methodology that operationalized NIS2 risk-management requirements through sensor-derived indicators and FMEA quantification. Three primary scientific contributions were achieved. First, the work extended the traditional Failure Mode and Effects Analysis (FMEA) model beyond hardware and reliability failures, incorporating cyber-induced failure modes affecting data integrity, availability, and authenticity in railway IoT and OT environments. Second, real-time anomaly indicators and streaming sensor evidence were integrated into dynamic RPN updates, enabling measurable risk variation tracking rather than static audit documentation. Third, the FMEA outputs were explicitly mapped onto regulatory obligations defined in NIS2 Article 21, demonstrating that compliance could be evidenced through data-driven KPIs such as detection latency, Mean Time to Detect (MTTD), and post-mitigation RPN contraction. Together, these contributions filled a methodological gap between governance frameworks and field-layer sensor analytics.

Despite these advances, several open challenges remained. The expert-driven scoring model and scenario-based anomaly evaluation limited reproducibility, particularly due to the absence of large-scale labelled datasets and inter-operator benchmarking. Cyber-threat evolution required periodic recalibration of the Occurrence (O) and Detection (D) parameters, as fixed expert estimates may lose validity over time without continuous evidence ingestion. Furthermore, integration of edge analytics into legacy OT systems demanded careful performance balancing, especially where safety certification cycles restrict frequent software changes. There also remained a methodological gap in linking RPN variation with resilience forecasting: while RPN reduction demonstrated control effectiveness retrospectively, its predictive interpretation required further empirical validation across broader railway environments.

Future work should therefore focus on dataset expansion across heterogeneous infrastructure, cross-operator validation of RPN dynamics under real incident conditions, and the development of federated learning pipelines that update detection models without exposing raw operational data. These efforts would strengthen the reproducibility of the proposed framework, progress toward standardized cyber-sensor benchmarks for transport infrastructure, and support the development of self-learning risk-management ecosystems consistent with the long-term objectives of NIS2 implementation.

## 8. Conclusions and Future Work

This study proposed and validated a sensor-centric framework for cybersecurity risk management in railway infrastructure, aligning the methodology with the requirements of the NIS2 Directive.

By extending the Failure Mode and Effects Analysis (FMEA) approach to encompass cyber-induced sensor failures, the research demonstrated how regulatory obligations can be operationalized through quantitative, data-driven indicators.

The main conclusions are as follows:

1. Sensor networks constitute the foundation of cybersecurity in cyber–physical railway systems. Their correct functioning and data integrity are essential for ensuring both operational safety and compliance with NIS2.
2. The adapted FMEA methodology effectively quantified cyber risks associated with sensor networks, providing structured prioritization based on severity, occurrence, and detection parameters.
3. Integration of real-time sensor monitoring enabled dynamic updates of RPN values, establishing a continuous feedback loop between risk assessment and control effectiveness evaluation—directly addressing NIS2 Criteria 1 and 6.
4. Machine learning and edge analytics significantly improved detectability, reducing detection latency and enabling predictive risk management.
5. The approach provided verifiable metrics (e.g., RPN, MTTD, DA, FAR) that can serve as objective evidence in cybersecurity audits and performance reviews mandated by competent authorities under NIS2.

Overall, the study demonstrated that railway cybersecurity cannot be confined to network protection or compliance documentation—it must extend to the sensor layer, where data originate and where operational resilience is determined in real time.

The proposed approach offers a practical mechanism for aligning technical risk assessment with governance-level compliance processes. By integrating FMEA results and sensor analytics into a unified management framework, railway operators can:

- Establish traceable links between technical incidents and NIS2 policy outcomes;
- Implement evidence-based evaluation of control effectiveness using sensor data;
- Support cross-functional coordination between safety, maintenance, and cybersecurity teams;
- Facilitate automated reporting and audit readiness through standardized performance indicators.

This integrated framework thus supports a transition from reactive compliance to proactive cyber resilience, where the railway system continuously validates its own security posture through sensor-derived intelligence.

Building upon the presented results, future research should focus on developing fully automated, self-adaptive risk management ecosystems for sensor-based critical infrastructure.

Key research and development directions include:

1. Digital Twins for Cyber Risk Assessment—creation of digital replicas of railway systems where simulated cyber events can be tested and mitigation strategies evaluated before deployment.
2. Integration with Blockchain Technologies—implementation of immutable audit trails for RPN evolution and event verification, ensuring full traceability of security control performance.
3. Adaptive Machine Learning Models—continuous retraining of algorithms using federated learning and transfer learning approaches to maintain detection efficiency across evolving threat landscapes.
4. Standardization of Sensor-Centric Cyber Metrics—development of harmonized indicators connecting NIS2 requirements with real-time data quality and integrity measurements.
5. Cross-Sector Applications—extending the proposed framework to other transport domains (aviation, maritime, road) and critical infrastructures where IoT systems play a similar role.

Due to the scenario-based and expert-driven nature of the study, the case-study network description does not include full implementation details such as exact ML architectures, hyperparameter grids, or resource benchmarks. The intention is to demonstrate how illustrative anomaly-detection mechanisms interact with FMEA scoring and the NIS2 process cycle, rather than to provide a hardware-specific or model-specific reproducibility package. A more detailed implementation study is planned as future work.

The paper does not include detailed network-topology diagrams, replay-path instrumentation, or raw log listings, as the aim is to present a governance-oriented and process-level analytical model rather than a network-performance benchmark. The latency values reported in the case-study scenario are intended to illustrate the impact of redundancy logic on the FMEA detectability dimension rather than to characterise system-level QoS.

In the long term, the fusion of sensing technologies, AI-driven analytics, and regulatory compliance frameworks will enable the creation of intelligent, self-monitoring infrastructure systems. Such systems will continuously evaluate their cyber posture and autonomously adapt to emerging threats—transforming compliance from a periodic obligation into an ongoing, sensor-driven capability.

The conducted research has shown that cybersecurity risk management in critical infrastructure must evolve from document-based frameworks toward evidence-based, data-centric systems.

By positioning sensors as active components of the cybersecurity architecture, the railway sector can not only meet regulatory obligations but also enhance the safety, reliability, and trustworthiness of its operations.

In conclusion, sensor-enabled cybersecurity governance—supported by structured methodologies such as FMEA and empowered by real-time analytics—represents a crucial step toward the digital resilience envisioned by the NIS2 Directive and the broader European strategy for secure, intelligent transport systems.

## Figures and Tables

**Table 1:** Risk Priority Numbers (RPN) calculated for representative cyber failure modes in railway sensor networks.

Sensor Type/Function	Failure Mode	S	O	D	RPN	Mapped NIS2 Criterion	Mitigation/Monitoring Approach
**Axle counter sensor**	Spoofing of train presence data	9	6	8	432	Criterion 1, 6	Implement data authentication and redundancy validation
**Track condition sensor**	Injection of false vibration data	8	7	7	392	Criterion 6	Introduce sensor data cross-correlation and AI-based anomaly detection
**Power distribution sensor**	Firmware tampering	10	4	9	360	Criterion 1	Secure boot, firmware integrity checks
**Environmental sensor**	Loss of connectivity (DoS attack)	7	8	6	336	Criterion 6	Redundant communication paths and frequency hopping
**Signaling equipment sensor**	Unauthorized calibration change	9	5	8	360	Criterion 1	Implement configuration management and access control policies
**Onboard diagnostics sensor**	Malicious data replay	8	6	9	432	Criterion 6	Timestamp verification, encrypted communication protocols

The above results illustrate how quantitative FMEA outputs (RPN values) can be directly used to prioritize cybersecurity improvements and to document compliance under the NIS2 framework. By periodically recalculating RPN values, railway operators can demonstrate continuous improvement of control effectiveness—a requirement explicitly stated in Article 21 (3).

**Table 2:** Representative subset of cyber failure modes and corresponding RPN values for railway sensor systems.

Sensor Function	Failure Mode	S	O	D	RPN	Risk Level	Mapped NIS2 Criterion
**Track vibration monitoring**	Spoofed sensor data injection	9	7	8	504	Unacceptable	Criterion 1, 6
**Axle counter**	False occupancy signal due to replay attack	10	6	8	480	Unacceptable	Criterion 1
**Power supply monitoring**	Firmware corruption via unsecured update	9	5	9	405	Unacceptable	Criterion 1
**Environmental sensing**	Network jamming (LoRaWAN gateway)	8	8	7	448	Unacceptable	Criterion 6
**Rolling stock diagnostics**	Malicious data replay to fleet database	8	7	8	448	Unacceptable	Criterion 6
**Signaling controller sensor**	Unauthorized configuration change	9	5	9	405	Unacceptable	Criterion 1
**Energy meter sensor**	Credential theft during transmission	7	7	6	294	Tolerable	Criterion 6
**Track temperature probe**	Physical tampering of sensor housing	6	5	6	180	Tolerable	Criterion 1
**Edge gateway node**	Malware infection via USB maintenance port	10	4	8	320	Unacceptable	Criterion 1
**Weather station node**	Unsecured API leading to data leakage	6	8	5	240	Tolerable	Criterion 6

This table presents a selected set of sensor-related cyber failure modes derived from the FMEA, illustrating how each identified vulnerability contributes to overall system risk under the NIS2 framework. The results indicate that the most severe risks correspond to data integrity and availability attacks, which directly influence train control and infrastructure monitoring. Among the top-ranked issues, spoofed sensor signals and firmware tampering scored RPN > 400, requiring immediate corrective actions.

**Table 3:** Key mitigation measures for the most critical sensor-level cyber failure modes identified in the FMEA.

Failure Mode	Recommended Mitigation Measure	Implementation Approach	Verification Indicator (KPI/KRIkri)
**Spoofed sensor data injection**	Sensor authentication and timestamp verification	Cryptographic signatures; redundant sensing nodes	% of validated packets; anomaly detection rate
**Firmware tampering**	Secure boot, digitally signed updates	Trusted Platform Module (TPM) verification	number of verified firmware updates/year
**Network jamming**	Frequency hopping, redundant gateways	Spectrum monitoring	Mean Time to Detect (MTTD) interference
**Unauthorized configuration change**	Role-based access control (RBAC), configuration integrity hashes	Access management platform	% of devices under configuration control
**Malware infection on gateway**	Port isolation, endpoint protection	USB device control policy	number of blocked unauthorized devices

These mitigations correspond directly to technical and procedural measures described in NIS2 Annex I (points a, c, f), linking the FMEA outputs with auditable evidence for compliance verification. The walkthrough presented in the case-study section serves as a conceptual process-level example. It demonstrates how sensor-derived indicators support the NIS2-required sequence of monitoring → detection → response → documentation, without prescribing a specific technical implementation. This aligns with the purpose of the article, which is to propose a governance-oriented analytical framework.

## Data Availability

The data presented in this study are available on request from the corresponding author.
